# Prevalence of dental caries, gingivitis and fluorosis among school children in rural schools: A cross-sectional study

**DOI:** 10.6026/973206300223609

**Published:** 2026-06-30

**Authors:** Debadrita Ghosh, Kolasani Balaram, Kanchan Malawat p, Shiprita Kumari, Saurabh Shekhar, Anagha Agrawal, Rahul Tiwari, Prashant M.C

**Affiliations:** 1 Department of Pediatric & Preventive Dentistry, Haldia institute of Dental Sciences & Research (HIDSAR), West Bengal, India; 2 Department of Dentistry, General Dentist, Aspen Dental, Manitowoc, Wisconsin, United States; 3 Department of Dentistry, Sardar Patel Medical College, Bikaner, Rajasthan, India; 4 Department of Human Anatomy, AIMST University, Bedong, Kedah, Malaysia; 5 Department of Oral Pathology and Microbiology, AIMST University, Bedong, Kedah, Malaysia; 6 Department of Public Health Dentistry, Narsinhbhai Patel Dental College and Hospital, Sankalchand Patel University, Visnagar, Gujarat, India; 7 Department of Oral and Maxillofacial Surgery, RKDF Dental College and Research Centre, Sarvepalli Radhakrishnan University, Bhopal, Madhya Pradesh, India

**Keywords:** Dental caries, gingivitis, dental fluorosis, rural school children, oral health epidemiology

## Abstract

Oral diseases such as dental caries, gingivitis and fluorosis remain highly prevalent among school children, particularly in rural populations where access to preventive dental care is limited. Therefore, it is of interest to assess the prevalence of dental caries, gingivitis and dental fluorosis among school-aged children attending rural schools through a cross-sectional epidemiological survey. A total of 300 school children aged 6-15 years were clinically examined using standardized indices including the DMFT index, gingival index and Dean's fluorosis index. Dental caries was the most common oral condition observed, followed by gingivitis and dental fluorosis, with notable variations according to age and gender. Thus, data shows the continued burden of preventable oral diseases in rural populations and emphasize the need for school-based oral health programs and preventive strategies.

## Background:

Oral health is an essential component of general health and significantly influences the quality of life, nutrition and psychological well-being of children. Among pediatric populations, dental caries, gingivitis and dental fluorosis are among the most commonly encountered oral health problems worldwide. Despite advances in preventive dentistry and public health initiatives, these conditions remain highly prevalent, particularly in rural and socioeconomically disadvantaged communities where access to oral health services and awareness regarding preventive practices are limited [1, 2]. Dental caries is considered the most common chronic disease affecting children globally. It is a multifactorial disease resulting from interactions between dental biofilm, fermentable carbohydrates, host susceptibility and time. Epidemiological studies have consistently reported a high prevalence of caries among school-aged children, with dietary habits, poor oral hygiene practices and limited fluoride exposure contributing to disease progression [3, 4]. In rural settings, the burden of dental caries is often higher due to reduced availability of dental services, lack of preventive programs and lower awareness among parents and children regarding oral hygiene practices [5]. Gingivitis represents the earliest and most reversible stage of periodontal disease and is commonly observed among children and adolescents. Gingival inflammation results primarily from plaque accumulation along the gingival margin and can progress to more severe periodontal conditions if left untreated. Several studies have indicated that gingivitis prevalence among school children remains significant, reflecting inadequate oral hygiene practices and limited access to preventive dental care [6, 7]. Early identification and management of gingivitis are essential to prevent the development of advanced periodontal disease later in life. Dental fluorosis is another condition frequently observed in children residing in regions with high fluoride concentrations in drinking water. Fluorosis results from excessive fluoride intake during the period of enamel development, leading to enamel hypomineralization characterized by discoloration and structural changes in tooth enamel. Although optimal fluoride exposure plays a crucial role in caries prevention, excessive exposure may lead to fluorosis, which can have aesthetic and psychological implications for affected individuals [8]. Several epidemiological studies conducted across different populations have reported varying prevalence rates of dental caries, gingivitis and fluorosis among children, reflecting differences in environmental exposure, dietary patterns, oral hygiene behaviors and socioeconomic conditions [9]. Rural populations, particularly in developing countries, often exhibit a higher prevalence of these conditions due to limited preventive dental services and lack of organized oral health promotion programs [10]. Therefore, it is of interest to assess the prevalence of dental caries, gingivitis and dental fluorosis among school children in rural schools through a cross-sectional clinical examination.

## Materials and Methods:

A cross-sectional epidemiological study was conducted to assess the prevalence of dental caries, gingivitis and dental fluorosis among school children studying in rural schools. The study was carried out over a period of six months after obtaining approval from the Institutional Ethics Committee and permission from school authorities. Informed consent was obtained from parents or guardians prior to examination. The study population consisted of school children aged between 6 and 15 years enrolled in rural government schools. A total of 300 children were included in the study using a convenience sampling method. Children who were present on the day of examination and whose parents provided consent were included in the study. Children with systemic illnesses, ongoing orthodontic treatment, or incomplete dentition due to trauma or congenital anomalies were excluded from the study. Clinical examinations were conducted within the school premises under natural daylight using sterile mouth mirrors and WHO periodontal probes. Standard infection control protocols were followed during the examination process. Dental caries was assessed using the Decayed, Missing and Filled Teeth (DMFT/dmft) index according to the criteria recommended by the World Health Organization. Gingival health status was evaluated using the Gingival Index, which measures the severity of gingival inflammation based on color, edema and bleeding on probing. Dental fluorosis was assessed using Dean's Fluorosis Index, which classifies fluorosis severity into categories such as normal, questionable, very mild, mild, moderate and severe based on enamel changes. Demographic details including age and gender were also recorded for each participant. All data were recorded in a standardized clinical examination form and subsequently entered into a spreadsheet for statistical analysis. Descriptive statistics were used to calculate the prevalence of dental caries, gingivitis and fluorosis among the study population. Frequencies and percentages were calculated for categorical variables. Statistical analysis was performed using SPSS software version 25.0.

## Results:

The study included a total of 300 school children aged between 6 and 15 years from rural schools. Clinical examination revealed that dental caries was the most prevalent oral health condition among the participants. Gingivitis was also frequently observed, particularly among older children who exhibited signs of poor oral hygiene. Dental fluorosis was present in a smaller proportion of children, likely reflecting variations in fluoride exposure within the community. Table 1 presents the demographic distribution of the study population according to age and gender. The majority of participants belonged to the 10-12-year age group (40.0%), followed by 13-15 years (33.3%) and 6-9 years (26.7%). Male participants (54.0%) slightly outnumbered females (46.0%). The distribution indicates adequate representation across different developmental stages. Table 2 shows the prevalence of dental caries based on the DMFT/dmft index. Dental caries was present in 168 children (56.0%), while 132 children (44.0%) were caries- free. This indicates a substantial burden of dental caries within the study population. The high proportion of untreated carious lesions suggests limited access to dental care services and inadequate awareness of preventive oral health practices among children and caregivers. Table 3 illustrates the prevalence of gingivitis among the participants. Gingivitis was observed in 142 children (47.3%), whereas 158 children (52.7%) showed healthy gingival status. The presence of gingival inflammation in nearly half of the study population highlights inadequate oral hygiene practices and underscores the need for early preventive interventions. Table 4 summarizes the distribution of dental fluorosis according to Dean's Fluorosis Index. The majority of participants (70.0%) exhibited normal enamel. Very mild fluorosis was observed in 14.0% of children, while 10.0% showed mild fluorosis. Moderate and severe fluorosis was relatively uncommon, affecting 4.7% and 1.3% of the participants, respectively. These findings suggest that although fluoride exposure exists in the region, most cases are of low severity and are unlikely to have significant clinical or aesthetic implications.

## Discussion:

Oral diseases remain among the most common chronic conditions affecting children worldwide, particularly in developing regions where preventive dental care and oral health awareness are limited. The present cross-sectional study evaluated the prevalence of dental caries, gingivitis and dental fluorosis among school children in rural schools and demonstrated a substantial burden of these oral conditions within the study population. The findings highlight the continuing public health importance of oral diseases among children living in rural communities. Dental caries was the most prevalent oral health condition observed in the present study, affecting more than half of the examined children. Similar findings have been reported in several epidemiological investigations conducted in different geographic regions. Alraqiq *et al.* reported that dental caries prevalence among school children remains considerably high despite improvements in preventive dental care programs, emphasizing that untreated carious lesions continue to represent a major global oral health problem in pediatric populations [11]. Likewise, Mittal *et al.* demonstrated that socioeconomic factors, dietary practices and oral hygiene behaviors significantly influence the development of dental caries among children, particularly in communities with limited access to preventive dental services [12]. The results of the present study are consistent with these findings and indicate that dental caries continues to represent a significant oral health challenge among rural school children. Another important finding of the present study was the high prevalence of gingivitis among the examined participants. Gingivitis was observed in nearly half of the children included in the study, indicating the presence of inadequate oral hygiene practices within the population. Gingivitis is considered the earliest stage of periodontal disease and is strongly associated with plaque accumulation and insufficient oral hygiene measures. Singh *et al.* reported that gingival inflammation among school children is commonly associated with poor oral hygiene habits, irregular tooth brushing practices and lack of awareness regarding oral health maintenance [13]. Similarly, Moothedath *et al.* reported that gingivitis prevalence among children and adolescents is influenced by multiple factors including oral hygiene practices, socioeconomic status and dietary habits [14]. The findings of the present study are comparable with these reports and suggest that improving oral hygiene education among school children may significantly reduce the burden of gingival diseases. Dental fluorosis was also observed among a proportion of children in the present study, although the majority of cases were categorized as mild forms. Dental fluorosis is primarily associated with excessive fluoride intake during the developmental stage of tooth enamel formation. Kumar *et al.* reported that mild forms of dental fluorosis are frequently observed in regions with elevated fluoride concentrations in drinking water, particularly in rural areas where groundwater serves as the primary source of drinking water [15]. Likewise, Hajare *et al.* demonstrated that the prevalence and severity of dental fluorosis are closely related to the fluoride concentration present in environmental sources such as groundwater and soil [16]. The relatively low prevalence of moderate and severe fluorosis observed in the present study suggests that fluoride exposure in the study region may be present at levels that are not excessively high but still capable of producing mild enamel changes. An interesting observation in the present study was the coexistence of dental caries and dental fluorosis in certain participants. This finding reflects the complex relationship between fluoride exposure and dental caries development. While fluoride plays an important role in preventing dental caries by enhancing enamel resistance to demineralization, excessive fluoride intake may result in fluorosis. Dong *et al.* reported that low to moderate fluoride exposure is associated with reduced dental caries prevalence but may simultaneously increase the likelihood of mild dental fluorosis [17]. Therefore, maintaining optimal fluoride concentrations in drinking water remains essential to balance the protective effects against dental caries while minimizing the risk of fluorosis. Socioeconomic and behavioral determinants also significantly influence oral health outcomes among children. Children residing in rural and economically disadvantaged communities often experience a higher burden of oral diseases due to limited access to dental care facilities and inadequate oral health awareness. Swain *et al.* reported that rural school children exhibited significantly higher levels of dental caries compared to their urban counterparts, primarily due to limited availability of preventive dental programs and reduced oral health literacy among parents and caregivers [18]. Similarly, Haresaku *et al.* emphasized that inadequate oral hygiene practices and lack of preventive education contribute significantly to the development of periodontal diseases among adolescents [19]. These findings reinforce the importance of implementing targeted oral health promotion strategies in rural communities. School-based oral health promotion programs have been widely recommended as an effective approach for improving oral health outcomes among children. Such programs provide opportunities for early detection of oral diseases, promotion of oral hygiene practices and dissemination of oral health education among children and teachers. Alayyan *et al.* reported that community-based and school-based oral health interventions can significantly reduce the prevalence of dental caries and improve oral hygiene behaviors among children when implemented consistently [20]. The findings of the present study further support the importance of implementing similar preventive programs in rural schools to address the burden of oral diseases among children. Overall, the results of the present study highlight the significant prevalence of dental caries, gingivitis and dental fluorosis among school children in rural schools. These findings emphasize the need for comprehensive preventive strategies including oral health education, dietary counseling, regular dental screening and fluoride monitoring programs. Implementing such interventions at the community and school level may play an important role in improving oral health outcomes among children residing in rural populations.

## Conclusion:

Dental caries, gingivitis and dental fluorosis remain prevalent oral health conditions among rural school children. Data shows the influence of oral hygiene practices, dietary habits and environmental fluoride exposure on pediatric oral health. Implementation of school -based oral health education and preventive programs is essential to reduce the burden of oral diseases in rural communities.

## Figures and Tables

**Table 1:** Age and Gender Distribution of Study Participants (n=300)

**Age Group (years)**	**Male**	**Female**	**Total**	**Percentage(%)**
6-9	42	38	80	26.7
10-12	65	55	120	40
13-15	55	45	100	33.3
Total	162	138	300	100

**Table 2:** Prevalence of Dental Caries (DMFT/dmft Index)

**Caries Status**	**Frequency**	**Percentage(%)**
Caries Present	168	56
Caries Absent	132	44
Total	300	100

**Table 3:** Prevalence of Gingivitis among Study Participants

**Gingival Status**	**Frequency**	**Percentage(%)**
Gingivitis Present	142	47.3
Gingivitis Absent	158	52.7
Total	300	100

**Table 4:** Distribution of Dental Fluorosis (Dean's Fluorosis Index)

**Fluorosis Category**	**Frequency**	**Percentage(%)**
Normal	210	70
Very Mild	42	14
Mild	30	10
Moderate	14	4.7
Severe	4	1.3
Total	300	100
